# Predicting longevity responses to dietary restriction: A stepping stone toward precision geroscience

**DOI:** 10.1371/journal.pgen.1008833

**Published:** 2020-07-09

**Authors:** Maria C. Perez-Matos, William B. Mair

**Affiliations:** Department of Molecular Metabolism, Harvard T. H. Chan School of Public Health, Boston, Massachusetts, United States of America; Princeton, UNITED STATES

Successes in public health during the last century have led to dramatic increases in human life expectancy worldwide. However, with this success has come a surge in age-related noncommunicable diseases (NCDs), including obesity, diabetes, and Alzheimer’s disease. Geroscience has evolved as a field focused on the prevention of such age-related NCDs, based upon the premise that the biology of aging is a shared risk factor that might be targeted to add healthy years of life to the elderly.

**Fig 1:**
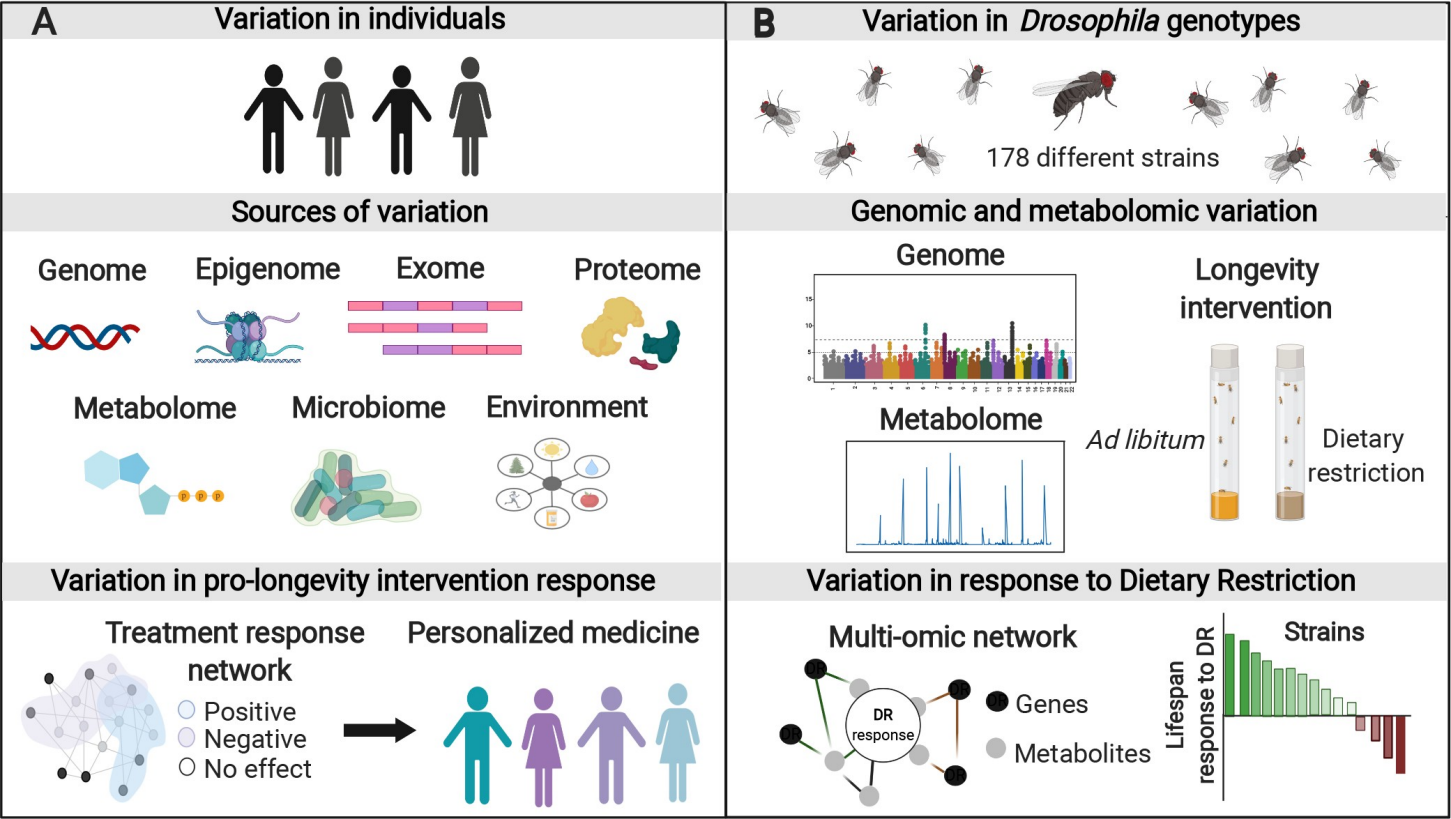
Multi-‘omic’s network-based approach towards precision geroscience. **(A)** Within population heterogeneity in the response to prolongevity interventions limits their therapeutic use. Multiple sources contribute to the variation in response to treatment: from genotype to microbiome, along with interaction with the environment. Information from all sources can be integrated in biological multi-‘omic’s networks that more accurately inform and predict an individual’s response to treatment as a pathway toward precision geroscience. **(B)** In an inbred, genetically heterogeneous population of fruit flies subject to DR, Jin and colleagues [11] integrated gene-level GWAS with metabolic profiling to build multi-‘omic’s network that display specific gene-to-metabolite-to-phenotype pathways directly associated with DR response. DR, dietary restriction; GWAS, genome-wide association study.

Targeting the mechanisms that underlie the prolongevity effect of dietary restriction (DR) (reduced food intake without malnutrition) remains the single most promising path toward interventions that might prolong healthy aging. For over 100 years, the effects of DR have been studied in controlled laboratory settings, largely focusing on a limited number of inbred strains available for each of the classical model organisms. However, there is increasing evidence of variation in the response to DR both within and between populations and genotypes [1–3]. This heterogeneity in response to treatment is expected to be equally present in human populations and represents a barrier that must be removed if we are to effectively translate geroscience to therapeutics. We will need to understand the molecular pathways that explain this phenomenon in order to develop individual-level strategies. Multiple layers of variation between individuals underlie variation in their response to antiaging therapeutics such as DR, with genotypic differences enhanced by differing environmental exposures. Changes to epigenetic, metabolite, and proteomic landscapes are both mediators and consequences of this gene-environment interaction, each one providing key insight into the underlying molecular process **(Fig 1A)** [4–8]. Although a number of DR-associated genes and metabolic signatures in response to DR have been identified [9,10], the specific cellular pathways that explain response heterogeneity remain to be elucidated.

In new work in this issue of *PLOS Genetics*, Jin and colleagues [11] aim to define predictors of the response to DR by integrating genetic and metabolomic information using a systems biology approach. They examine the DR response in 178 inbred strains of *Drosophila melanogaster*. As expected, the response of the different strains to DR was heterogeneous; while most strains lived longer with DR, some showed no response at all, and, in a third group DR shortened life span. To begin to define mechanisms behind this variation in response, they identified the metabolome landscape associated with increased longevity in response to DR, using response to treatment, relative life span (rLS) on DR versus *ad libitum* (AL) rather than median life span, as the outcome. This unique and innovative approach allows the identification of metabolites that are highly correlated with life span extension, rather than life span per se. In accordance with previous reports, DR remodeled the metabolome in a consistent way. From 105 metabolites analyzed, abundance of 24 individual metabolites correlated with rLS. In addition, when the authors looked for changes in abundance of metabolites between DR and AL fed animals, the predictive list narrowed further, with only 4 metabolites associating with rLS. To study the metabolome as a dynamic structure, Jin and colleagues identified covariance of metabolites with longevity. They found global network structural changes in response to diet; some connections were gained, others lost, and identified a number of “hub” metabolites that might putatively explain variation in response to treatment. Positive hits included alpha-ketoglutarate and glutamine, known to be associated with life span [12].

To achieve their ultimate goal of identifying candidate gene-to-metabolite-to-phenotype pathways of the DR response, the authors obtained significance scores from multiple individual gene-level GWAS, using metabolites as quantitative traits. Based on these scores, they built connections between genes, metabolites, and response phenotype, producing a network that depicts associations formed in response to treatment as well as gene-metabolite groups. The edges of the network and how they change in response to treatment can provide more comprehensive information compared to assessing genes or metabolites in isolation **(Fig 1B)**. Of note, variance in the CCHamide-2 receptor (CCHa2R), a neuropeptide receptor involved in satiety, correlated both with levels of the hub metabolites and rLS. Using an inducible RNAi system to further empirically test the effects of this and other gene hits on response to DR, CCHa2R knockdown was shown to increase response to treatment, increasing life span during DR but not during AL feeding.

This work represents an initial but critical step in moving geroscience away from population-based responses toward precision aging therapeutics, with the means to predict an individual’s response to treatment. Using metabolomic profiling and network modeling on inbred, genetically heterogeneous populations enabled the identification of relevant pathways that explain variation in DR-mediated life span extension in fruit flies. Additionally, RNA interference (RNAi) validation supported the role in longevity of pathways identified using this approach. Just as with any medical intervention, translating geroscience discovery to human therapeutics for NCDs will require more than a one-size-fits-all approach. The next steps in the field will be to apply this network analysis approach to optimize dosage and predict genotype-specific longevity responses for other treatment candidates, such as rapamycin. In addition, it will be informative to study whether predictive factors for the rLS response to DR in *Drosophila* are conserved in mammals. As a field, our common aim is to have enough objective information about genes by environment and treatment interactions in order to develop predictive strategies that allow for personalized medicine approaches to optimize healthy aging in humans. Although we have a long way to go, Jin and colleagues outline the beginning of a roadmap geroscience can follow to translate the basic science of aging biology to usable therapies for the elderly.
